# Humanoid robot intervention vs. treatment as usual for loneliness in long-term care homes: Study protocol for a pilot randomized controlled trial

**DOI:** 10.3389/fpsyt.2022.1003881

**Published:** 2022-10-21

**Authors:** Paola Lavin, Myriam Lesage, Edward Monroe, Michael Kanevsky, Johanna Gruber, Karin Cinalioglu, Soham Rej, Harmehr Sekhon

**Affiliations:** ^1^Department of Psychiatry, GeriPARTy Research Group, Jewish General Hospital, Montréal, QC, Canada; ^2^Awakening Health Limited, Hong Kong, Hong Kong SAR, China; ^3^CONNECTIN-TeleEmpathy™, Village Medical Center™, Global Health University™, Montréal, QC, Canada; ^4^Department of Psychiatry, Jewish General Hospital, McGill University, Montréal, QC, Canada; ^5^Department of Psychiatry, McLean Hospital and Harvard Medical School, Cambridge, MA, United States

**Keywords:** humanoid robot, loneliness, long-term care, depression, stress

## Abstract

**Background:**

Loneliness affects up to 42% of long-term care residents and is associated with poor health outcomes. Humanoid robot interventions hold promise for reducing loneliness and decreasing barriers to social interaction in long-term care settings, such as the current COVID-19 safety measures in many countries, limited mobility, and poor health. We present a protocol describing an assessor-blinded randomized controlled trial comparing the effects of a humanoid robot intervention to treatment as usual, on loneliness and mental health outcomes in long-term care residents.

**Methods:**

Seventy-four (*n* = 74) older adults experiencing loneliness in 3 long-term care homes will be randomized 1:1 to an 8-week, twice a week social intervention with the Grace humanoid robot vs. a treatment as usual active control. We will assess change (baseline to week 8) in (1) loneliness (primary outcome), (2) depression severity, and (3) stress (secondary outcomes), as well as (4) other exploratory outcomes: anxiety, quality of life and reduction in acute healthcare utilization. We will also assess the feasibility and acceptability of the intervention using qualitative methods.

**Discussion:**

The proposed study will assess the effects of a social robot on loneliness and other mental health outcomes, as well as the feasibility of the intervention in older adults living in long-term care settings.

**Trial registration:**

NCT05423899.

## Introduction

Loneliness in long term care homes (LTC) is a worldwide epidemic, affecting up to 42% of the resident population compared to 10% of community-dwelling older adults (1), resulting in over $6.7 billion in direct costs annually in the U.S. alone (2). Loneliness is associated with poor mental health outcomes, such as increased stress, anxiety, and poor quality of life, and is considered both a symptom and a trigger for mental health conditions such as depression. Older adults are particularly vulnerable to loneliness due to circumstances associated with aging, such as losses of relationships, bereavement, medical comorbidities, retirement, decline in physical and cognitive function, and changes in living environments (3).

Mental health conditions in older adults are often managed with pharmacological treatment, with over 25% of lonely older adults being prescribed medications such as sedatives, opioids, antidepressants, and benzodiazepines (4). Pharmacological treatments are ineffective in up to 50–80% of older adults (5) and are often accompanied by adverse effects leading to discontinuation in as many as 30% of individuals (6). Psychotherapies, such as talk therapy, can be used as adjuncts or alternatives to pharmacotherapy (7) but require specialized therapists who are challenging to access, with waiting lists up to 12 months in Canada (8). These interventions are also administered one-on-one, which makes it a human-resource intensive and costly intervention. Loneliness in LTC homes is sometimes mitigated through recreational and group activities, however due to low staff-to-resident ratios as well as funding shortages and social distancing restrictions, these activities are often limited and the first to be defunded (9). There is thus an urgent need to test potentially effective, acceptable, and feasible behavioral interventions.

Social robots targeting loneliness are a promising intervention with growing evidence of having positive effects in older adults' mental health (10, 11). However, there is limited research on human-like robot interventions in older adults; over 70% of studies focus on pet robots, and only 11% on social/humanoid robots (12). Studies focusing on socially assistive humanoid robots have been mainly conducted in individuals with dementia (13, 14). Participants developed feelings of trust toward the robot, experienced reduced anxiety following interactions with the robot (13) and reported positive attitudes toward a long-term companionship with the robot (14). These case studies included ≤ 10 participants and did not include control groups. While preliminary findings are promising, there are no RCTs investigating the effects of a humanoid robot intervention on mental health outcomes in older adults. This study will assess the effectiveness, acceptability, and feasibility of the Grace humanoid robot vs. treatment as usual (TAU; group activities in the LTC) on symptoms of loneliness, stress and depression, as well as anxiety, quality of life and reduction in acute healthcare utilization.

## Methods

### Study design

This is a two-arm, assessor-blinded Randomized Controlled Trial (RCT) with 74 planned participants, examining an 8-week, 2 sessions per week, 30-min/session humanoid robot interaction intervention vs. treatment as usual (TAU) control group, in older adults who experience loneliness living in three LTC homes in Montreal, Quebec and Ottawa, Ontario, Canada.

### Interventions

#### Grace robot

The Grace robot was designed by Awakening Health/Hanson Robotics for healthcare settings and to interact with the elderly and those isolated by the COVID-19 pandemic (15). Grace is a robot with a human-like appearance, which can move, actively listen, engage in conversation and react appropriately to human emotions. Intervention activities will mostly consist of active listening and general discussions about topics of interest (e.g., hobbies, music). The participant will also have the option of other types of interactions with the robot, including robot-led meditation, robot-led light exercise, listening to music and singing. Because loneliness is a subjective experience and does not have a standardized solution, this is a personalized intervention approach (16). We anticipate that each older adult participant will have different needs and wishes when interacting with the robot, which will allow for a more natural interaction with the robot.

#### Treatment as usual

The treatment as usual active control group will not receive the robot intervention. We have deliberately chosen the participating LTC homes due to their high frequency of social interactions for their clients as part of their routine care (e.g., one-on-one and group activities, family interaction, exercise groups) compared to most LTC homes settings, making TAU an active control.

### Participants

We will recruit 74 participants from three LTC homes in Montreal and Ottawa in 12–15 months. The inclusion and exclusion criteria for participation in this study are outlined in Table 1.

**Table 1:** Eligibility criteria.

**Inclusion criteria**	**Exclusion criteria**
(1) Aged ≥60 years old	(1) Do not speak English
(2) Living in a LTC home setting in Montreal	(2) Inability to provide consent
(3) Cognitively healthy, have mild cognitive impairment (MCI) or mild dementia (MMSE score of >20/clinical opinion of LTC homes staff members)	(3) Moderate to severe dementia (MMSE score <18/diagnosis of moderate-severe dementia/clinical opinion of LTC homes staff members)
(4) Able to provide consent	(4) Significant hearing loss
(5) Loneliness UCLA-3 (17) score of ≥ 6 or more (moderate-severe loneliness).	(5) Acutely unstable medical illnesses, including delirium, acute cerebrovascular/cardiovascular events within the last 6 months; having a terminal medical diagnosis with prognosis of < 12 months
	(6) High suicide risk (e.g., active suicidal ideation and/or current/recent intent or plan).

#### Randomization

Participants will undergo stratified 1:1 randomization [strata: site and baseline cognition (normal vs. MCI/dementia)] to the intervention or active control group to account for unequal numbers of participants and differential severity of cognitive impairments between sites. To maintain allocation concealment, participants will be asked to refrain from disclosing their allocation into either the intervention group or the treatment-as-usual group to other residents until data collection is completed.

### Outcomes

All primary, secondary, and exploratory data will be collected at baseline and 8-weeks (primary study endpoint). The total time required to complete each time point assessment is 30–40 min.

#### Primary outcome

Loneliness as measured by the Revised UCLA 20-item Loneliness Scale (18) at baseline vs. 8–weeks (primary study endpoint), a commonly used validated scale to screen for loneliness.

#### Secondary outcomes

Secondary Outcome A: Stress, as measured by the Perceived Stress Scale [PSS; (19)], a 14-item scale used to measure the degree to which life events are experienced and appraised as stressful.

Secondary Outcome B: Depression, as measured by the Patient Health Questionnaire [PHQ-9; (20)], a 9-item self-report questionnaire used to diagnose depression and assess symptom severity.

#### Exploratory outcomes

Anxiety and quality of life, as measured by Generalized Anxiety Disorder-7 (GAD-7) (21), EQ-5D-5L (22), respectively. We will also look for indications of a reduction in acute healthcare utilization (number of hospitalizations and emergency room visits) at 8 weeks post-intervention. Moreover, participant experiences and acceptability with the robot will be measured using qualitative methods (semi-structured interviews). We will also note whether feasibility outcomes have been met, wherein a) >50% of eligible participants will consent, b) recruitment goals are met during the study period (*n* = 74), and c) rate of attrition is <20% (dichotomous “yes/no”).

### Statistical analyses

Descriptive statistics will be run with continuous variables summarized using means and standard deviations, and categorical variables summarized using counts and proportions. Primary analyses for all quantitative outcomes including the primary, secondary and exploratory outcome measures (e.g., UCLA loneliness score, PHQ-9 score, GAD-7 score) will be compared between intervention and control groups, controlling for baseline scores using linear mixed models (SAS Institute, Cary, NC). For qualitative data, thematic analysis will identify and code central themes, using NVivo software (ver.10).

#### Power analysis

We estimate a total sample size of 74 participants recruited and randomized, with 60 study completers, taking into account a likely 20% attrition rate (loss to follow-up, not tolerating the intervention) based on our previous experience with RCTs (23–25). In this study, 74 participants will be randomized to either the treatment group or control group, with each having *n* = 37. On repeated measures ANOVA, 60 study completers will allow us to observe an effect size of 0.34 at two-tailed alpha = 0.05 and Power (1-Beta) = 0.8 (cite G^*^Power software). As this is a pilot study, results will be used for power calculations in order to assess an appropriate sample size for a future larger confirmatory RCT.

#### Subgroup analyses

##### Sex-based subgroup analysis

We will complete a subgroup analysis reporting main outcomes in men and women, respectively. Sex, along with other baseline characteristics, will also be included as covariates in statistical models to capture their effects on outcomes. We will conduct subgroup analyses on primary outcome in patients stratified by site.

## Discussion

The proposed study is the first RCT investigating the effects of a humanoid robot intervention on mental health outcomes in older adults. Low staff-to-resident ratios as well as funding shortages and social distancing restrictions in LTC homes have underscored the urgent need for effective, acceptable, and feasible interventions to address loneliness in this setting. Artificial Intelligence (AI)-based humanoid robots, such as the Grace robot, can provide the complex interactions required for human-like social interaction, can foster connections, and engage participants to promote positive health outcomes (26, 27). Humanoid robots may also decrease barriers to social interaction in LTC homes, such as limited mobility and poor health. This is especially relevant during COVID-19 and any infectious-disease or immunocompromised case, as participants can experience social interaction without increasing their risk of infection (28). The proposed study investigating the effect of a humanoid robot interaction intervention vs. TAU on loneliness and mental health outcomes will be the first of its kind in Canada. If successful, this study will pave the way for humanoid robots as a novel approach to promote aging in place, which is likely to become increasingly cost-effective in the coming years. Furthermore, humanoid robots have the potential to become an accessible and scalable alternative to loneliness interventions in LTC settings. A recent report from the Stanford Institute for Human-Centered Artificial intelligence determined that costs of robots are steadily decreasing as AI investments continue to climb worldwide and AI capabilities improve (29).

The potential benefits of the proposed pilot include decreased loneliness, anxiety, depression, and other improved mental health outcomes in older adults, as well as decreased healthcare utilization and improved overall quality of life. The results from this cutting-edge study will provide pilot data to inform a larger three-arm, multi-site confirmatory RCT assessing the effects of the Grace robot on mental health outcomes of older adults in LTC homes.

## Ethics statement

Ethical approval was not provided for this study on human participants because Research Ethics Board Approval has not yet been requested but will be sought at the following sites: CIUSSS-Center-Ouest-de-l'Ile; CIUSSS-Ouest-de-l'Ile in Montréal, QC. Informed consent will be obtained from all study participants by trained research staff at all sites in accordance with these sites' respective governing ethics boards' guidelines. Written informed consent was not provided because recruitment has not yet started. Written informed consent will be requested from all participants.

## Author contributions

Conceptualization and methodology: PL, EM, MK, SR, and HS. Draft preparation and writing and editing: ML, PL, HS, and SR. Project administration and management: PL, ML, EM, MK, HS, and SR. Funding acquisition: ML, KC, PL, HS, JG, and SR. All authors have substantially contributed to the preparation, critical review, commentary revision, and approval of the manuscript.

## Conflict of interest

SR receives a salary award from the Fonds de Recherche de Québec Santé FRQS is a consultant for AbbVie and a shareholder of Aifred Health. HS has a CIHR fellowship award, MITACS fellowship award, and AGE-WELL award. MK is affiliated with CONNECTIN and subsidiaries is partnered with Awakening Health and consults globally for public and private sector organizations to heal a fragmented medical system. EM was employed by Awakening Health Limited. The remaining authors declare that the research was conducted in the absence of any commercial or financial relationships that could be construed as a potential conflict of interest.

## Publisher's note

All claims expressed in this article are solely those of the authors and do not necessarily represent those of their affiliated organizations, or those of the publisher, the editors and the reviewers. Any product that may be evaluated in this article, or claim that may be made by its manufacturer, is not guaranteed or endorsed by the publisher.
